# Exploring the Osteogenic Potential of Zinc-Doped Magnesium Phosphate Cement (ZMPC): A Novel Material for Orthopedic Bone Defect Repair

**DOI:** 10.3390/biomedicines12020344

**Published:** 2024-02-01

**Authors:** Yinchu Liu, Ling Yu, Jingteng Chen, Shiyu Li, Zhun Wei, Weichun Guo

**Affiliations:** Department of Orthopedics, Renmin Hospital of Wuhan University, Wuhan 430060, China

**Keywords:** bone defect, zinc-doped magnesium phosphate cement, bone repair, osteogenic differentiation, rat bone marrow mesenchymal stem cells

## Abstract

In orthopedics, the repair of bone defects remains challenging. In previous research reports, magnesium phosphate cements (MPCs) were widely used because of their excellent mechanical properties, which have been widely used in the field of orthopedic medicine. We built a new k-struvite (MPC) cement obtained from zinc oxide (ZnO) and assessed its osteogenic properties. Zinc-doped magnesium phosphate cement (ZMPC) is a novel material with good biocompatibility and degradability. This article summarizes the preparation method, physicochemical properties, and biological properties of ZMPC through research on this material. The results show that ZMPC has the same strength and toughness (25.3 ± 1.73 MPa to 20.18 ± 2.11 MPa), that meet the requirements of bone repair. Furthermore, the material can gradually degrade (12.27% ± 1.11% in 28 days) and promote osteogenic differentiation (relative protein expression level increased 2–3 times) of rat bone marrow mesenchymal stem cells (rBMSCs) in vitro. In addition, in vivo confirmation revealed increased bone regeneration in a rat calvarial defect model compared with MPC alone. Therefore, ZMPC has broad application prospects and is expected to be an important repair material in the field of orthopedic medicine.

## 1. Introduction

Millions of people suffer from bone defects due to tumor onset, trauma, or bone-related diseases around the world [1]. Although natural bone tissues possess inherent capabilities for self-recovery through self-healing processes in response to minor damage, extensive bone fractures or significant defects often make it challenging to achieve complete functional recovery without surgical intervention [2]. The regeneration of bone defects remains a major clinical challenge. Various bone grafts such as autografts and allografts have been employed to repair and regenerate bone defects [3]. However, there are many disadvantages such as the limited availability of autogenous bone and the risk of donor site morbidity [4]. Synthetic biomaterials play pivotal role in tissue engineering.

In the mid-1980s, Brown et al. invented calcium phosphate bone cement (CPC) [5], however, the simple use of CPC materials has major shortcomings, namely, low early strength and slow strength development. Therefore, it is necessary to further improve the performance of CPC materials [6].

Magnesium phosphate cements have been investigated in recent years as alternatives to calcium phosphate cements for bone repair applications [7]. Magnesium phosphate bone cement is characterized by its rapid setting and high early strength properties. The heat release rate during the cement hydration process is controllable, and the curing volume undergoes slight expansion. This allows for a more secure integration with bones, promoting a robust embedding connection. The cement exhibits high biocompatibility, making it well-suited for applications in the treatment of unstable fractures and the adhesive fixation of artificial joint prosthetics. Additionally, its excellent plasticity further contributes to its versatility. These superior qualities collectively contribute to the widespread potential applications of magnesium phosphate bone cement in the fields of unstable bone fracture treatment and the fixation of artificial joint implants [8,9]. As the fourth most common mineral in the human body, magnesium is involved in hundreds of biochemical reactions and is an important element in the development of bones and soft tissues [10]. Healthy adults can regain 24–30 g of Mg to maintain normal function, and the recommended daily allowance for Mg maintenance is 310–420 mg. Excess magnesium ions are allowed because they can be transported through the circulatory system and quickly excreted through urine and face without causing any adverse effects [11]. Therefore, magnesium-based metals can be defined as a new class of absorbable metal materials [12].

Zinc (Zn), one of the key trace elements in bone tissues, comprises only a small fraction of bone minerals and is widely acknowledged as crucial for bone metabolism [13]. It has been reported to stimulate the osteogenic differentiation of stem cells [14,15]. Xia et al. constructed C-ZnO nanocarbon-modified fibrous scaffolds for stem cell-based osteogenic differentiation [16]. Chandramohan et al. recommended that growth of MSCs can be successfully attained with CS/PCL/Zn scaffolds [17]. Extracellular Zn ion concentration was found to modulate the osteogenic differentiation of a human immortalized cranial periosteal cell line [14]. In recent years, Zn-containing materials have been widely applied in the fields of biomedicine, biotechnology, and analytical chemistry [18,19,20]. Zn is a component or activator of approximately 300 enzymes or their isoforms and therefore significantly impacts the functioning of various human body areas. It is the only metal constituent of all six classes of enzymes [21], serves as a cofactor for alkaline phosphatase (ALP), and participates in bone tissue metabolism [22]. Studies have indicated that zinc plays a significant role in bone tissue formation in in vitro experiments with osteoblasts [23,24]. Seo et al.’s research demonstrated that zinc treatment of osteoblastic MC3T3-E1 cells affected their proliferation, collagen synthesis, and the activity of the bone marker protein alkaline phosphatase (ALP) [25]. Furthermore, human studies indicate that oral administration of zinc at a dose of 3060 µmol/kg can increase alkaline phosphatase (*ALP*) activity and the DNA content of bone tissue [26,27].

Throughout life, the bone is in a dynamic balance involving a complex coordination of multiple bone marrow cell types. It is estimated that in the adult human body, the entire skeleton is renewed every seven years [28,29]. In the bone marrow, mesenchymal stem cells represent a minute subset of nucleated cells, comprising 0.001–0.01% of the total nucleated cell population, and osteoblasts are derived from bone marrow mesenchymal stem cells (BMSCs). Therefore, inducing differentiation of BMSCs in the osteogenic direction is crucial for the reconstruction of bone defects [30].

Many osteogenic molecules reside in the bone and have the potential to mediate fracture or defect repair. Bone morphogenetic protein 2 (*BMP2*) is a necessary component of the inherent regenerative capacity of the bone and provides the first molecular insight into the initiation of endogenous skeletal repair [31]. ALP is one of the first functional genes identified to be involved in calcification. At least one of its functions in the mineralization cycle is also expected to take place at an early stage [32]. The osteogenic differentiation of MSCs is regulated by Runt-related transcription factor 2 (*Runx2)* and Osterix, which sequentially activate osteo-promoting signaling pathways, such as transforming growth factor–β (TGF-β) and wingless-related integration site (Wnt)/β-catenin [33]. Furthermore, numerous bone matrix protein-coding genes, such as osteopontin (*OPN*) and osteocalcin (*OCN*), are target genes of Runx2 and Osterix (Osx) [34,35]. The above genes play an important role in the differentiation of MSCs into osteogenesis. This article also initially explores the osteogenic mechanism of these materials.

In summary, we aimed to develop a new zinc-doped magnesium phosphate bone cement with the goal of inducing the osteogenic differentiation of mesenchymal stem cells by adjusting the concentration of zinc, thereby repairing critical bone defects. The formulation of ZMPC as a novel bone repair material retains the high compressive strength of the original MPC system while effectively promoting the osteogenic differentiation of MSCs. In our study, we demonstrated the potential of ZMPC to enhance bone regeneration by evaluating its physicochemical properties and surface morphology and conducting in vitro and in vivo experiments. Experiments assessing the ability of the materials to regulate the adhesion, proliferation, and osteogenic differentiation of rBMSCs confirmed that ZMPC promotes both spontaneous and induced osteogenesis. This suggests the possibility of improving bone regeneration.

## 2. Materials and Methods

### 2.1. Materials

MgO; purity 98.5% (Sinopharm Chemical Reagents, Shanghai, China); KH2PO4; purity 99.5% (Sinopharm Chemical Reagents, Shanghai, China); ZnO (4N, Sinopharm Chemical Reagents, Shanghai, China).

### 2.2. MPC and ZMPC Preparation

The solid phase of the MPC consisted of magnesia and potassium dihydrogen phosphate with a molar ratio of 3:2. MgO was calcinated at 1600 °C for 4 h. This composite material underwent grinding in a ball mill and was subsequently sieved using a 200-mesh screen to achieve particles of approximately 75 μm. To prepare the composite bone cement, zinc oxide (ZnO; 4N, Sinopharm Chemical Reagents, Shanghai, China) was introduced to deionized water and thoroughly stirred. The solid and liquid phases were then meticulously mixed at a powder/liquid ratio (P/L) of 2 (g/mL), resulting in a homogeneous paste that was transferred to cylindrical molds. Subsequently, all samples were incubated at 37 °C under 100% relative humidity for 72 h before further experiments were conducted. The sample compositions of the samples are listed in Table 1.

### 2.3. Chemical Composition and Surface Morphology

The hardened bone cement was ground into powder and X-ray diffraction analysis (XRD; Rigaku intelligent X-ray diffractometer, SmartLab SE, Akishima-shi, Tokyo Metropolis, Japanese) was performed to assess the composition over a 2θ range of 10–80° using Cu–K α radiation. A chemical composition analysis was performed using a Raman spectrometer (XploRA plus, HORIBA Jobin Yvon, France). Raman shift range: 60–8000 cm^−1^. Excitation light: 532 nm, 100 mW. The surface morphologies of the cement samples were observed using scanning electron microscopy (SEM; Carl Zeiss, Cambridge, UK). Accelerating voltage: 0.1–30 kV. Probe current: 4 pA–20 nA.

### 2.4. Physicochemical Properties: Compressive Strength, Degradation, Mg^2+^ Release, pH Variation, Setting Time and Porosity

The scaffolds (diameter = 8 mm, height = 1 mm) were subjected to vacuum drying and the initial mass was recorded. Subsequently, the scaffolds were immersed in 0.05 M Tris buffer (pH = 7.4) at a concentration of 200 mg/mL. The buffer solution was refreshed every 2 days, and the scaffolds were weighed and recorded after washing with PBS and vacuum drying at the designated time points (1st, 3rd, 5th, 7th, 14th, 21st, and 28th days). Next, 100 μL of solution was removed at the indicated time points and the concentration of magnesium ion (Mg^2+^) measured with an atomic absorption spectrophotometer (ContrAA700; Analytik Jena, Jena, Germany), and the pH value of the medium measured using a pH meter (PHS-25; INESA, Shanghai, China). The compressive strength of the samples (diameter = 8 mm, height = 1 cm) was measured at a loading rate of 1 mm/min using a universal testing machine (SHT4 605, MTS Systems, Minneapolis, MN, USA) according to the standards proposed by the British Standards Authority.

The solidification time was defined as the time taken before a needle could not penetrate 1 mm into the sample using a Vicat instrument according to the Chinese National Standard (GB/T1346-2011) [36]. 

Cement porosity was measured after curing for 7 days using Archimedes’ principle. Briefly, the samples were soaked in absolute ethanol until saturated. The weights of the samples before and after soaking with alcohol were denoted W1 and W2, respectively, and the suspended weight of the sample soaked in ethanol was denoted W3. The porosity calculation was as follows:

P (%) = (W2 − W1)/(W2 − W3) × 100%

### 2.5. In Vitro Studies

#### 2.5.1. Cell Proliferation and Adhesion

The sample eluate was prepared in accordance with the ISO10993-12 standard [37]. Briefly, sterile scaffolds (Disinfection by soaking in 75% alcohol, followed by overnight exposure to ultraviolet light (XYT, Suzhou, ZW8S24Y):185 nm, 20 mW/cm^2^) were immersed in complete culture medium at a concentration of 200 mg/mL for 72 h. Following centrifugation (1200 rpm/min), the supernatant was collected and stored at 4 °C.

The mesenchymal stem cells were isolated from the Sprague Dawley rat bone marrow of Sprague Dawley rats (rMSCs, STCC5011P, Zeesan Biotech, Xiamen, China). The cells were grown in a humidified atmosphere containing 5% CO_2_ at 37 °C. All samples were sterilized prior to cell seeding. Then cells were seeded in the well, and co-cultured with the extraction solution for one, three and five days. Next, a 10 μL cell counting kit-8 (CCK-8; Dojindo Laboratories, Kumamoto, Japan) was incubated with the cells for 2 h at 37 °C. The optical density was measured at 450 nm using a microplate reader. The viability of MSCs was evaluated using live/dead staining (Invitrogen, Eugene, OR, USA). After being cultured with leaching liquids for five days, 500 μL of the live/dead reagent was added to each well. An inverted fluorescence microscope (Olympus IX71, Tokyo, Japan) was used to examine the live and dead cells. The adhesion area was quantified using the ImageJ software (1.8.0, NIH, Bethesda, MD, USA). Three random areas were used to obtain average values.

After co-culturing the cells with the material for 48 h, the samples underwent a meticulous process involving washing, fixation in 4% paraformaldehyde, dehydration through graded alcohol, oven drying, and subsequent examination via SEM after vacuum gold sputter coating (Carl Zeiss, Cambridge, UK).

#### 2.5.2. Quantitative Real-Time Polymerase Chain Reaction

Total RNA was synthesized using TRIzol (Invitrogen; 15596026) reagent and the RevertAid™ first-strand cDNA synthesis kit (Thermo Scientific, Norristown, PA, USA; K1622). Real-time polymerase chain reaction (PCR) was performed using an ABI PRISM 7900HT sequence detection system (Applied Biosystems, Foster City, CA, USA). The expression of osteogenesis-related genes was normalized to GAPDH using the 2^−ΔΔCT^ method. Primer sequences are listed in Table 2.

#### 2.5.3. Western Blotting

Proteins were extracted from cell or tissue samples using a protein extraction buffer and subsequently separated by SDS-PAGE. Following electrophoresis, the proteins were transferred onto a polyvinylidene difluoride membrane using the wet transfer method. The membrane was then blocked with 5% non-fat milk to prevent nonspecific binding and subsequently probed with a specific primary antibody targeting the protein of interest overnight at 4 °C. After thorough washing with TBST to remove the unbound primary antibody, the membranes were incubated with a secondary antibody for 1 h at room temperature. The ECL kit (Servicebio Technology) was used to visualize the membranes. The sources of the primary antibodies were as follows: GAPDH (Mouse, 60004Ig, 1:10,000, Proteintech, Wuhan, China), BMP2 (Mouse, A0231, 1:2000, Proteintech, Wuhan, China), OCN (Rabbit, A20800, 1:1000, ABclonal, Wuhan, China), RUNX2 (Rabbit, AF4770, 1:2000, Affinity, Jiangsu), ALP (Rabbit, AF4770, 1:2000, Affinity, Shanghai, China). The source of the secondary antibody is as follows: HRP-Goat anti Rabbit (Searcare, 5220-0336, 1:50,000), HRP-Goat anti Mouse (Searcare, 5220-0341, 1:50,000).

#### 2.5.4. Immunofluorescence Microscopy

Cells (4 × 10^4^) were cultured in confocal dishes, fixed with 4% formaldehyde for 20 min, and permeabilized with 0.1% Triton X-100 for 20 min. After blocking with 5% BSA (in TBST), the cells were incubated with the primary antibodies against OPN (boster, Wuhan, PB0589, 1:200) and ALP (absin, Shanghai, abs125381, 1:200) at 4 °C overnight. Secondary antibodies were then incubated with the cells at room temperature for 30 min, and DAPI was applied to stain the nuclei. The source of the secondary antibody is as follows: Anti-Goat IgG (H + L) Antibody, Peroxidase-Labeled (SeraCare, Milford, MA, USA, 5220-0362, 1:400); Anti-Rabbit IgG (H + L) Antibody, Peroxidase-Labeled (SeraCare 5220-0336, 1:400); Anti-Mouse IgG (H + L) Antibody, Human Serum Adsorbed and Peroxidase-Labeled (SeraCare, US, 5220-0341, 1:400); Anti-Rat IgG (H + L) Antibody, Peroxidase-Labeled (SeraCare, US, 5220-0364, 1:200).

Immunofluorescence (IF) images were captured using a confocal microscopy (Olympus, Tokyo, Japan).

### 2.6. In Vivo Experiments

#### 2.6.1. Construction of Animal Models

**Figure 1:**
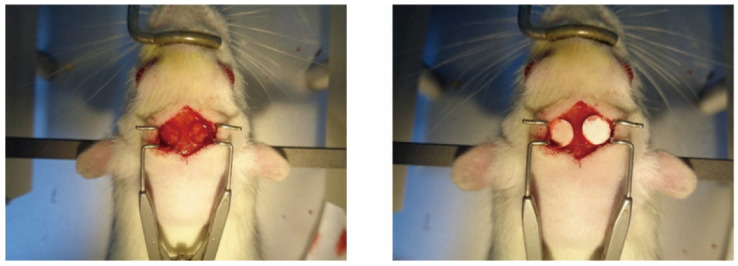
Image of rat skull defect.

SD rats aged 8–10 weeks were randomized into three groups (*n* = 6) to undergo bilateral calvarial bone defect surgery. Group 1: no cement (control); group 2: MPC; group 3: ZMPC. They were weighed, and anesthesia was induced by intraperitoneal injection of ketamine (100 mg/kg) and xylazine (10 mg/kg). Rats were then secured using a stereotaxic apparatus. The skin over the cranial area was disinfected with iodine, and a 2.0 cm mid-sagittal incision was made in the center of the scalp to expose the skull. Using a cranial drill, bilateral symmetrical cranial defects, each with a diameter of 6 mm, were created on both sides of the midline, corresponding to the rat’s ear line and cross-stitch areal. Sterile saline was used continuously to cool the drill during drilling. Care was taken to avoid damaging the meninges during drilling. The drilling process was monitored, and when it felt like penetration was imminent, the bone chips were carefully lifted using forceps. If needed, drilling and lifting were performed simultaneously, resulting in 6 mm defects on both sides of the skull. After completing the drilling for each group of rats, the corresponding regenerative materials were implanted bilaterally into the defects. The skin over the cranial area was then sutured and disinfected with iodine, and the rats were placed on a warming pad until their body temperature recovered before being returned to their cages for continued care (Figure 1).

#### 2.6.2. Micro-Computed Tomography

Micro-CT scans were performed after fixing all the sampled specimens. The scanning parameters were as follows: X-ray tube current of 200 uA, voltage of 85 KV, scanning of the entire object, scanning resolution of 10.156007 μm, exposure time of 384 ms, and a scanning angle of 180 degrees. A phantom scan (Phantom; provided with uniform configuration by the equipment manufacturer) was performed under the same conditions for calibration. Rraw images were acquired after scanning.

The region of interest (ROI) was analyzed using CT Analyzer software (version 1.20.3.0, Bruker Corporation, Bermen, Germany). By setting consistent parameters, the software computed various metrics, including the total volume (TV), bone volume (BV), volume fraction (BV/TV), bone surface area (BS), trabecular number (Tb.N), trabecular separation (Tb.Sp) and trabecular thickness (Tb.th), providing comprehensive insights into tissue characteristics.

#### 2.6.3. Histological Analysis

The dissected calvariae were fixed and embedded in paraffin without decalcification. They were then dehydrated in a series of ethanol solutions with increasing concentrations, and each dehydration step lasted 40 min. Subsequently, the samples were immersed in three containers filled with xylene until they became transparent, followed by immersion in three containers containing with liquid paraffin. Finally, the dehydrated tissue blocks were embedded in a mold filled with liquid paraffin. Embedding was performed using a hard tissue microtome (Leica SP1600; Leica Biosystems, Nussloch, Germany). The specimens were washed five times during the dewaxing. They were then stained with hematoxylin and eosin (H&E) or Masson’s trichrome. Observations and imaging were conducted under a microscope, and digital images were captured using a digital camera (Olympus BX51; Olympus, Tokyo, Japan).

### 2.7. Statistical Analysis

Statistical analysis was performed using Prism 9.0 statistical analysis software. All data are expressed as mean ± SEM unless otherwise indicated. Data were analyzed using a one-way or two-way analysis of variance (ANOVA), followed by Student’s *t* test as appropriate, and *p* values are indicated by asterisks as follows: * *p* < 0.05, ** *p* < 0.01, *** *p* < 0.001, *p* < 0.05 was deemed to indicate statistical significance.

## 3. Results

### 3.1. Morphological and Physicochemical Characterization of the MPC and ZMPCs

#### 3.1.1. Field Emission Scanning Electron Microscopy (FE-SEM) Examination

**Figure 2:**
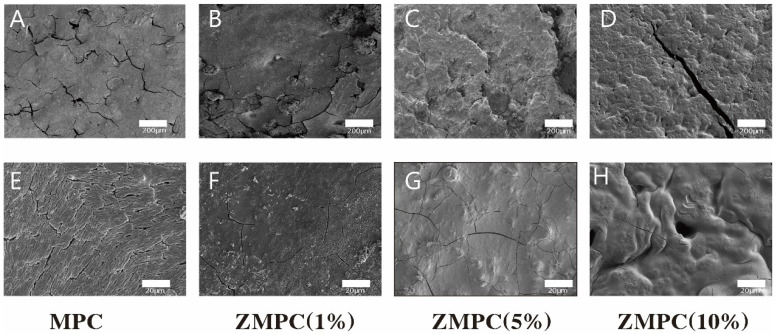
(**A**–**H**) SEM images of the surface morphology of different cements.

To further reveal the apparent mechanism of the composites at the microscopic scale, FE-SEM was performed to examine the morphologies of MPC and ZMPC as shown in Figure 2. Comparing the morphology and structure of MPC in the control group, it can be seen that at 100×, the cracks of ZMPC with zinc mass fractions of 1% and 5% were significantly reduced, while at 1000×, the hydrates formed by the reaction of ZMPC with 5% and 10% zinc made the surface more patchy, which may be more conducive to cell adhesion.

#### 3.1.2. XRD and Raman Examination

**Figure 3:**
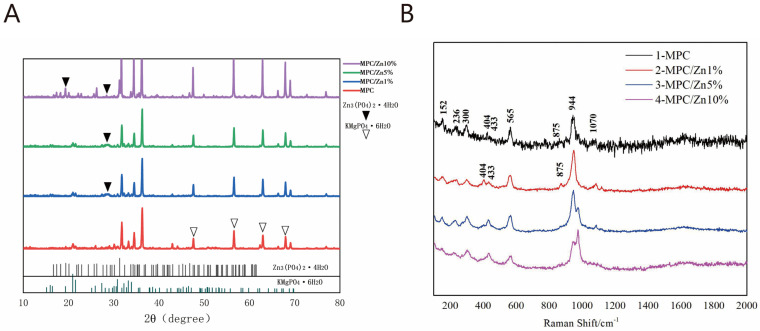
XRD patterns (**A**) and Raman (**B**) of different composites.

The XRD patterns of MPC and ZMPCs are shown in Figure 3A. The major hydraulic product was essentially similar which was potassium magnesium phosphate hexahydrate (KMgPO_4_·6H_2_O). Furthermore, the characteristic peaks of the zinc phosphate groups became more pronounced as the percentage of Zn increased.

The Raman spectrum data of magnesium phosphate and doped zinc were baseline-processed, normalized and drawn using Origin software to obtain the Raman spectrum as shown in the Figure 3B. Through Raman spectroscopy, it can be observed that there are distinct Raman vibrational peaks at 152 cm^−1^, 236 cm^−1^, 300 cm^−1^, 404 cm^−1^, 433 cm^−1^, 875 cm^−1^, 944 cm^−1^, and 1070 cm^−1^. Raman spectroscopy typically provides information on phosphate groups in five different frequency ranges. In the range of 1025 cm^−1^ to 1080 cm^−1^, the main vibrational mode is O-P-O bond bending. In the range of 800 cm^−1^ to 970 cm^−1^, the primary mode is P-O bond symmetric stretching. The range of 560 cm^−1^ to 615 cm^−1^ corresponds to O-P-O bond bending. In the range of 400 cm^−1^ to 480 cm^−1^, the main mode is P-O bond asymmetric stretching, whereas frequencies below 400 cm^−1^ mainly arise from lattice vibrations. Therefore, in the control group MPC, the vibrational peaks at 152 cm^−1^, 236 cm^−1^, and 300 cm^−1^ correspond to the lattice vibrations of magnesium phosphate; the peaks at 404 cm^−1^ and 433 cm^−1^ correspond to P-O bond asymmetric stretching vibrations; the peak at 565 cm^−1^ corresponds to the O-P-O bond bending mode; and the peaks at 875 cm^−1^ and 944 cm^−1^ correspond to P-O bond symmetric stretching, whereas the peak at 1070 cm^−1^ corresponds to O-P-O bond bending. Upon adding Zn to the sample, compared to the control group, the Raman vibrational peak at 875 cm^−1^ disappears. This may be attributed to the interaction between zinc and phosphate groups after the addition of ZnO, which suppresses the symmetric stretching mode of the P-O bond. This suggests that with an increase in the Zn content after the addition of ZnO, there is a certain level of interaction with phosphate groups.

#### 3.1.3. Mechanical and Physicochemical Properties of the Scaffolds

**Figure 4:**
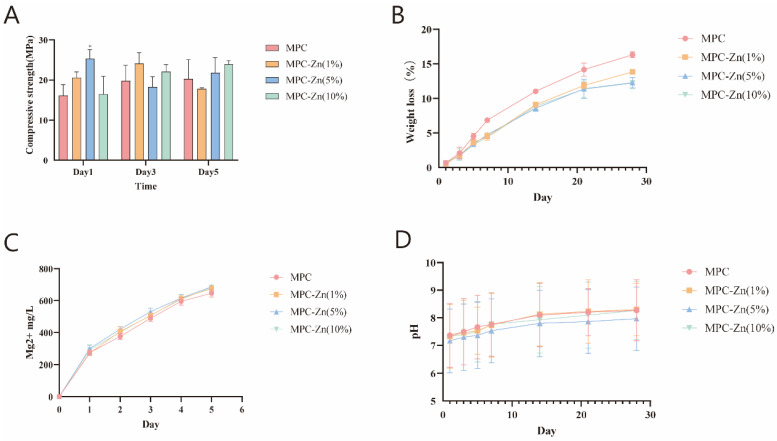
(**A**) Compressive strength. (**B**) Weight loss. (**C**) Mg^2+^ released into medium. (**D**) pH of immersion medium.

By examining the comparative analysis depicted in Figure 4A, it is evident that the introduction of zinc yields a notable enhancement in the compressive strength of MPC, particularly reaching 25.3 ± 1.73 MPa in the 5% group. As the curing duration extends, the inter-group disparities remained relatively marginal. The compressive strength remains at approximately 20 MPa.

The incorporation of Zn did not exert a discernible influence on Mg^2+^ release and pH, as shown in Figure 4C,D. Surprisingly, the incorporation of Zn resulted in a marginal decrease in the degradation rate of the composite material. With an increasing zinc mass fraction, the degradation rate decreased from 16.3% ± 1.08% in the control group to 13.83% ± 1.41%, 12.27% ± 1.11%, and 12.2% ± 0.92% in Figure 4B. The interaction between the zinc and phosphate groups enhanced the overall stability of the system, making it more resistant to degradation.

### 3.2. In Vitro Experiments on the MPC and ZMPCs

#### 3.2.1. Cell Proliferation and Adhesion on Samples

**Figure 5:**
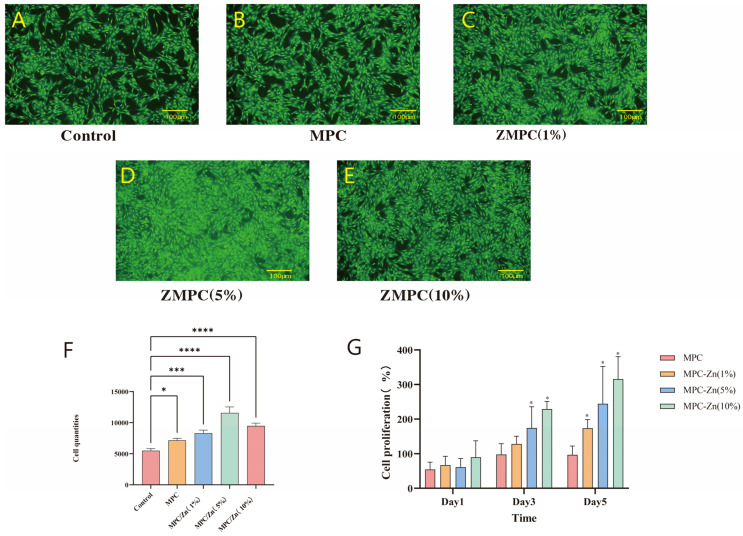
(**A**–**E**) Live/dead assays of BMSCs. (**F**) Quantitative analysis of Live/dead assays. (**G**) CCK8 analysis of BMSCs at day 1, 3 and 5, respectively. * *p* < 0.05, *** *p* < 0.001, **** *p* < 0.0001.

To ensure the successful use of the materials in our intended applications, a live/dead assay was used to evaluate cell viability. Viable cells were visible with few cells near the samples showing red fluorescence, indicating dead cells. After 72 h of co-culturing mesenchymal stem cells with the material, as observed in Figure 5A–E, we can observe that the cell proliferation in the ZMPC (5%) group was relatively high, whereas in the ZMPC (10%) group, it decreased. This indicates that when the Zn mass fraction is elevated to a certain extent, there may be a certain degree of cytotoxicity. The CCK-8 experimental results in Figure 5 are generally consistent with the aforementioned findings, and the optical density (OD) values indicate that the proliferation rate of mesenchymal stem cells improved after the addition of zinc, with the most significant enhancement observed in the 5% concentration group.

#### 3.2.2. Osteogenic Differentiation Behaviors

**Figure 6:**
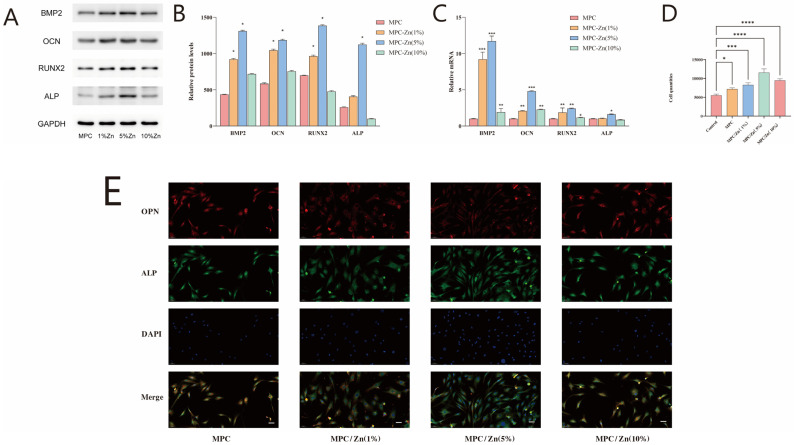
(**A**,**B**) Osteogenic differentiation on the samples. The osteogenic gene markers BMP2, OCN RUNX2 and ALP were determined using quantitative PCR and western blotting. (**C**,**D**) Quantitative analysis of western blotting and fluorescence intensity. (**E**) Immunofluorescence images stained with OPN (red) and ALP (green). The scale is 50 μm. * *p* < 0.05, ** *p* < 0.01, *** *p* < 0.001,**** *p* < 0.0001.

We examined the influence of various composite materials on the osteogenic differentiation of MSCs by evaluating the expression of four pivotal marker genes: RUNX2, BMP2, OCN, and ALP. After culturing cells on the composite material for 7 and 14 days, the expression of osteogenic markers was comprehensively analyzed through quantitative PCR and Western blotting. Our study revealed that compared to the MPC group, ZMPC significantly enhanced the expression of osteogenic proteins, with a more pronounced effect in the 5% group. Notably, elevated expression of BMP2 was particularly significant. (Figure 6A,B)

ALP is a hallmark enzyme of osteogenesis, and its expression appears early in the process of bone formation, indicating that the cells are undergoing osteogenic differentiation. OPN is a protein involved in bone tissue formation and remodeling, and its expression occurs during the mid-stage of osteogenic differentiation. Immunofluorescence staining showed that the expression levels of ALP and OPN were higher in MSCs cultured with 1% and 5% zinc mass fractions in the ZMPC group than those in the MPC and control group (Figure 6E). This was confirmed through quantitative analysis (Figure 6D), consistent with previous cell proliferation experiment results. This suggests that appropriate concentration of Zn promotes the expression of osteogenic proteins.

#### 3.2.3. In Vitro Osteogenesis of Samples

**Figure 7:**
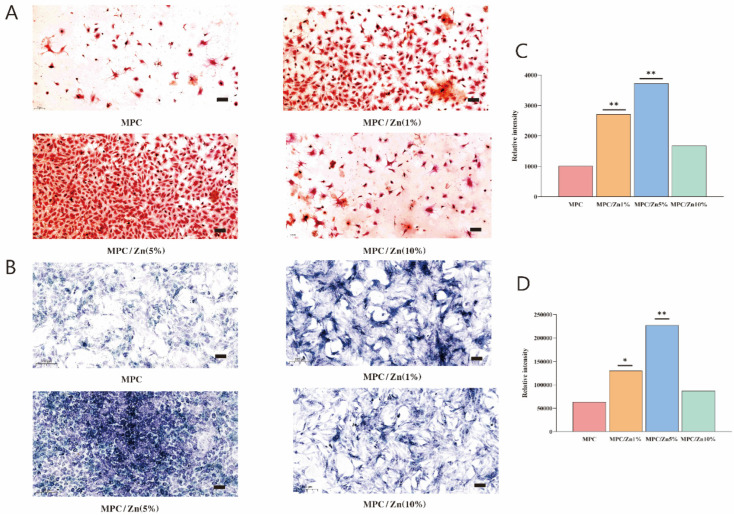
(**A**) ARS staining of BMSCs for 21 days. (**B**) ALP staining of BMSCs for 14 days. (**C**,**D**) Quantitative analysis by imageJ, * *p* < 0.05, ** *p* < 0.01.

ALP is one of the earliest genes expressed in the process of bone formation. After 14 days of co-culture with MSCs, ALP staining showed that the expression level of ZMPC (5%) was significantly higher than that in the other groups, indicating its potential to induce MSC osteogenic differentiation. To further determine the calcium deposition rate of MSCs under the same conditions, co-culturing was conducted for 21 days and assessed through alizarin red staining, consistent with the results of ALP-related assays. The ZMPC (5%) group expressed a more mineralized matrix and exhibited significantly higher levels of calcium deposition compared to the control group (Figure 7).

### 3.3. In Vivo Experiments on the MPC and ZMPCs

#### 3.3.1. Micro-CT Analysis

**Figure 8:**
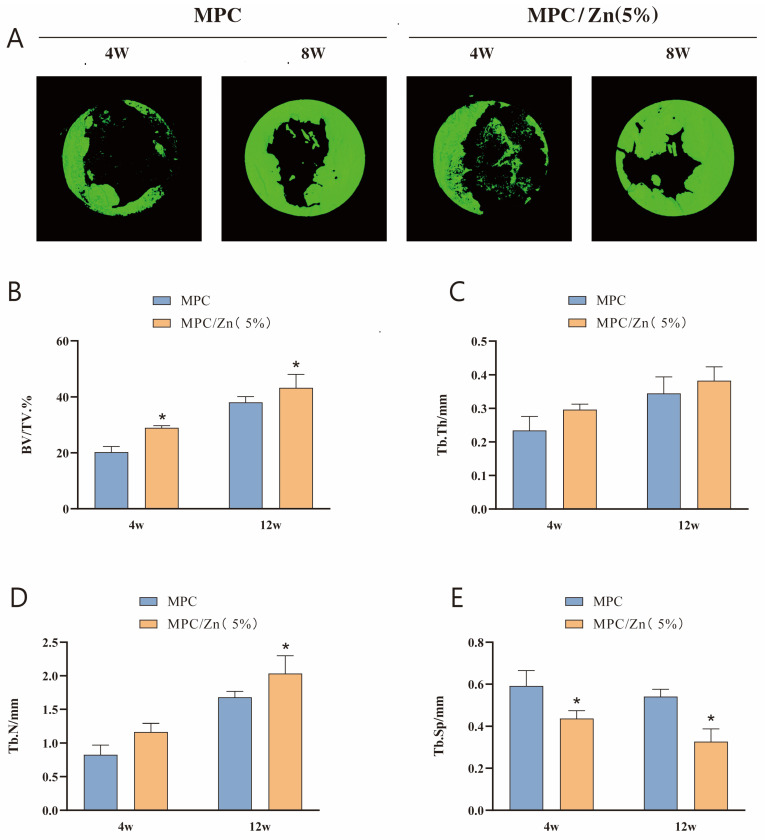
(**A**,**B**) Micro-CT reconstruction of calvarial defects after 4 and 12 weeks. (**C**–**E**) Quantitative analysis of BV/TV, Tb.N, Tb.th and Tb.Sp, * *p* < 0.05.

Micro-CT technology was employed to assess the volume of the newly formed bone in the defect. Four weeks postoperatively, micro-CT images of both ZMPC and MPC groups revealed scattered regenerated bone tissue. Twelve weeks post-implantation, the ZMPC group exhibited a significantly larger bone formation area than the MPC group (Figure 8A,B). Results were evaluated using Micro-CT parameters at different time points for each group. After 4 weeks, the new bone volume in the ZMPC group (20.28 ± 2.01%) was significantly greater than that in the control group (28.97 ± 0.73%). However, at 12 weeks post-transplantation, the ZMPC group showed significantly higher BV/TV and Tb.N values compared to the MPC group (43.29 ± 4.79% vs. 38.07 ± 2.08%, 2.03 ± 0.26 mm vs. 1.68 ± 0.23 mm); the ZMPC group’s Sp value was significantly lower than that of the MPC group (0.33 ± 0.06 mm vs. 0.54 ± 0.03 mm). These results indicate that ZMPC strongly promotes calvarial healing in rats.

#### 3.3.2. Histological Analysis

**Figure 9:**
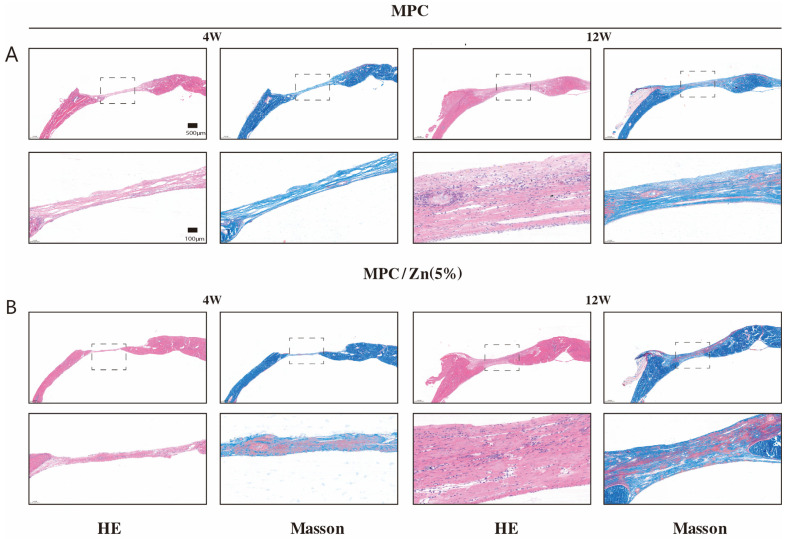
H&E and Masson staining of non-decalcified rat cranial sections of regenerated bone observed at 4 and 12 weeks following implantation. (**A**) MPC (**B**) MPC/Zn (5%).

At 4- and 12-weeks post-implantation, the bone-implant interface at the rat calvarial defect sites in the MPC and ZMPC groups was evaluated using H&E and Masson’s trichrome staining. H&E staining revealed no signs of inflammation, necrosis, or infection at the transplant site, indicating high biocompatibility of the bone cement. Both MPC and SM-35 demonstrated bone regeneration ability at four weeks, as evidenced by the initiation of bone callus formation. However, some connective tissue persisted at the defect site, and the ZMPC group exhibited a denser osseous structure. Twelve weeks post-implantation, both groups demonstrated new bone formation histologically. Notably, the ZMPC group exhibited more extensive development of trabeculae and blood vessels than the MPC group. Conversely, the MPC group showed a higher proportion of fibrous tissue. These findings indicate that, in comparison to the use of MPC alone, the incorporation of zinc into bone cement can enhance the repair of severe bone defects (Figure 9) Supplementary Materials Figures S1 and S2.

## 4. Discussion

Whole bone strength is determined not only by bone mass, but also by bone morphology and microarchitecture [38]. The unique aspect of the skeleton lies in its organic structure, which hardens through the integration of minerals, enabling it to provide support for movement and protect internal organs [39]. When osteoblasts deposit on a collagenous bone matrix, hydroxyapatite crystals accumulate, leading to the formation of mineralized bone [40]. This process consists of two stages: rapid initiation (primary mineralization), followed by slower mineral accumulation (secondary mineralization), which continues until it reaches the maximum level or the skeleton undergoes remodeling for renewal. During bone formation, osteoblasts integrate into the non-mineralized bone matrix and differentiate into bone cells [41]. In accordance with the characteristics of bone formation, we attempted to use the synthesized ZMPC as a cell deposition carrier to achieve native mineralization.

In our previous studies on MPC modification [42], we primarily utilized biologically active substances such as chitosan and sodium alginate, which formed relatively loose chemical bonds. After studying the composite using XRD and Raman spectroscopy, it was observed that ZnO is incorporated into the MPC system, partially forming zinc phosphate. Electron microscopy results also indicated a more cohesive integration. However, the Zn content was too low, making it challenging for our current methods to accurately and stably measure its release concentration. This limitation hindered further exploration of the effect of zinc concentration on osteogenic differentiation, and we only conducted simple measurements of a few common osteogenic gene markers. Osx is a human protein encoded by the SP7 [43]. It is a member of the zinc finger transcription factor Sp family and is highly conserved in osteogenic vertebrate species [44]. Sp7 plays a crucial role in driving bone formation along with Runx2 and distal-less homeobox (Dlx5) [45]. It is involved in the differentiation of MSCs into osteoblasts, ultimately leading to their differentiation into bone cells. Therefore, in future studies, we will closely investigate the relationship between this gene and zinc ions, with the aim of a more in-depth exploration of the underlying mechanisms.

The cranial defect model has many advantages such as the biological inertness of the cranial bone, standardized defect size, sufficient surgical access, and support for the implantation of biomaterials because of the presence of the dura mater and covering skin. Based on the physicochemical properties of the cement and in vitro responses, MPC/Zn (5%) was chosen for in vivo assessment of its reparative capabilities in a rat calvarial defect model, with MPC serving as a control. By employing HE staining, we observed a richer population of osteoblasts in the ZMPC group at the tissue level than in the control group. This observation aligns with the in vitro experiments showing elevated expression of osteogenic genes in BMSCs. Masson’s trichrome staining further provided detailed information about the collagen and fibrous tissue. The ZMPC group exhibited enhanced differentiation capabilities, manifested by fewer fibrous components and denser newly formed bone tissue. Quantitative analysis using microCT corroborated these observations, accurately illustrating the changes in rat calvarial bone in terms of volume, density, and microstructure. This comprehensive multi-level analysis revealed the promotional effects of ZMPC at the bone tissue level, providing a profound understanding of its potential applications in bone defect repair. This study provides robust support for future research and clinical practice.

## 5. Conclusions

By incorporating zinc oxide into a magnesium phosphate cement system, we successfully developed a novel bone repair material. For the MPC system, the introduction of zinc oxide did not significantly decrease the mechanical strength. More importantly, an appropriate concentration of zinc can promote cell proliferation and osteogenesis. Furthermore, a rat cranial bone defect model demonstrated the enhanced osteogenesis and bone ingrowth effects of the novel ZMPC. Collectively, the novel ZMPC offer promising prospects for future clinical applications.

## Figures and Tables

**Table 1:** The basic component ratios of the sample.

Samples	ZnO (wt%)
MPC	0
MPC/Zn(1%)	1.25
MPC/Zn(5%)	6.23
MPC/Zn(10%)	12.46

**Table 2:** Primer sequences used in quantitative PCR assay.

Primer	Sequence (5′ to 3′)
R-GAPDH-F	AGACAGCCGCATCTTCTTGT
R-GAPDH-R	CTTGCCGTGGGTAGAGTCAT
	
R-ALP-F	GAGGCTGAACCGCAGGATGT
R-ALP-R	GTCAATACCGGAAGGAGTGCT
	
R-OCN-F	GCAGACCTAGCAGACACCAT
R-OCN-R	TTGGACATGAAGGCTTTGTCA
	
R-RUNX2-F	GAGCACAAACATGGCTGAGA
R-RUNX2-R	TGGAGATGTTGCTCTGTTCG
	
R-BMP2-F	ATATGCTCGACCTGTACCGC
R-BMP2-R	TCCTCGATGGCTTCTTCGTG

## Data Availability

All the data sets used and/or analyzed in our study and Supplementary Materials are available.

## Supplementary Materials

The following supporting information can be downloaded at: https://www.mdpi.com/article/10.3390/biomedicines12020344/s1, Figure S1. The porosity of the materials; Figure S2. The injectability of the materials.
