# Supplementary figures and images for: Exploring the Osteogenic Potential of Zinc-Doped Magnesium Phosphate Cement (ZMPC): A Novel Material for Orthopedic Bone Defect Repair
Source: Biomedicines. 2024 Feb 1;12(2):344. doi: 10.3390/biomedicines12020344 (PMC10886858; doi:10.3390/biomedicines12020344)

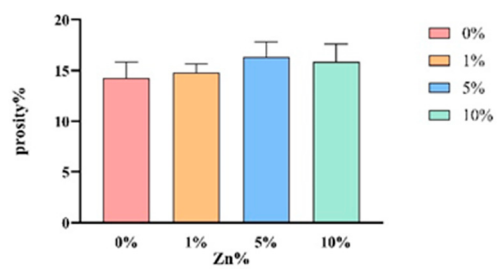

**Figure S1.** The porosity of the materials.

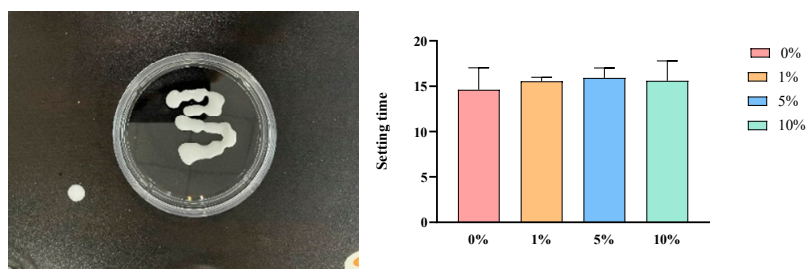

**Figure S2.** The injectability of the materials.

Supplement: Supplementary file 1 [file biomedicines-12-00344-s001.zip › biomedicines-2781829-supplementary.pdf]
